# Significance of Ubiquitin Carboxy-Terminal Hydrolase L1 Elevations in Athletes after Sub-Concussive Head Hits

**DOI:** 10.1371/journal.pone.0096296

**Published:** 2014-05-07

**Authors:** Vikram Puvenna, Chanda Brennan, Gerald Shaw, Cui Yang, Nicola Marchi, Jeffrey J. Bazarian, Kian Merchant-Borna, Damir Janigro

**Affiliations:** 1 Cerebrovascular Research, Cleveland Clinic Lerner College of Medicine, Cleveland, Ohio, United States of America; 2 Department of Neurosurgery, Cleveland Clinic Lerner College of Medicine, Cleveland, Ohio, United States of America; 3 Department of Molecular Medicine, Cleveland Clinic Lerner College of Medicine, Cleveland, Ohio, United States of America; 4 Department of Clinical-Bioanalytical Chemistry, Cleveland State University, Cleveland, Ohio, United States of America; 5 Departments of Emergency Medicine and Neurosurgery, University of Rochester Medical Center, Rochester, New York, United States of America; 6 Department of Neuroscience, University of Florida, Gainesville, Florida, United States of America; Biological Research Centre of the Hungarian Academy of Sciences, Hungary

## Abstract

The impact of sub-concussive head hits (sub-CHIs) has been recently investigated in American football players, a population at risk for varying degrees of post-traumatic sequelae. Results show how sub-CHIs in athletes translate in serum as the appearance of reporters of blood-brain barrier disruption (BBBD), how the number and severity of sub-CHIs correlate with elevations of putative markers of brain injury is unknown. Serum brain injury markers such as UCH-L1 depend on BBBD. We investigated the effects of sub-CHIs in collegiate football players on markers of BBBD, markers of cerebrospinal fluid leakage (serum beta 2-transferrin) and markers of brain damage. Emergency room patients admitted for a clinically-diagnosed mild traumatic brain injury (mTBI) were used as positive controls. Healthy volunteers were used as negative controls. Specifically this study was designed to determine the use of UCH-L1 as an aid in the diagnosis of sub-concussive head injury in athletes. The extent and intensity of head impacts and serum values of S100B, UCH-L1, and beta-2 transferrin were measured pre- and post-game from 15 college football players who did not experience a concussion after a game. S100B was elevated in players experiencing the most sub-CHIs; UCH-L1 levels were also elevated but did not correlate with S100B or sub-CHIs. Beta-2 transferrin levels remained unchanged. No correlation between UCH-L1 levels and mTBI were measured in patients. Low levels of S100B were able to rule out mTBI and high S100B levels correlated with TBI severity. UCH-L1 did not display any interpretable change in football players or in individuals with mild TBI. The significance of UCH-L1 changes in sub-concussions or mTBI needs to be further elucidated.

## Introduction

Concussions are a frequent occurrence among contact sport athletes, estimates vary in the range of 1.6–3.8 million per year [1]. However, the often neglected sub-concussive head blows (sub-CHIs) are even more common. Sub-CHIs have been defined as impacts that are not of a magnitude sufficient to cause a clinically diagnosable concussion yet sufficient to cause detectable changes [2], [3]. Initially described among boxers, sub-CHIs occur frequently among athletes involved in football, soccer, and hockey [4]–[6]. There is a recognized and urgent need for diagnostic and prognostic markers of mild traumatic brain injury (mTBI) as seen in football players and military personnel or civilians after road accidents or other types of harmful events. The quest for peripheral markers of mTBI has been confounded by several factors, including the inconsistency in the definition of concussive or subconcussive head hits and the presence of a blood-brain barrier (BBB) separating the brain from the systemic circulation which is tapped to determine the presence or levels of brain-derived signals [7], [8].

The cerebrovascular blood-brain barrier which consists of endothelial cells characterized by tight junctional contacts allowing restricted paracellular permeability [9]. Traumatic brain injury is often accompanied by blood-brain barrier disruption (BBBD) [10]. Thus, measurement and diagnosis of head trauma may rely on two markers, a marker of cerebrovascular integrity and one of brain injury [7]. Markers of brain damage are usually proteins shed by neuronal or glial cells undergoing pathological changes. These include neuron-specific enolase (NSE), ubiquitin carboxy-terminal hydrolase L1 (UCH-L1) and glial fibrillary acidic protein (GFAP). These markers, however, have never been investigated in conjunction of other markers of CNS insults, for example markers of blood-brain barrier disruption.

An obstacle in clinical BBB research on is the lack of sensitive measures of its integrity. In a clinical setting, BBBD can be determined radiologically (contrast enhanced MRI scans). This approach essentially consists of measuring the level of extravasation of a paramagnetic marker (gadolinium) injected intravenously. Conversely, lumbar punctures can be used to determine the albumin quotient between low cerebrospinal fluid levels and normally several-fold higher blood albumin levels. Serum markers of BBBD are molecular reporters that move across the BBB in the opposite direction of contrast agents or serum albumin. Thus, when BBB permeability is impaired, gadolinium will enter the CNS along with albumin while peripheral BBBD reporters will exit by the same paracellular pathway. Specific blood tests such as S100B has allowed for the measurement of BBB function [11]. S100 proteins [12] are small acidic proteins belonging to the largest Ca^+2^ binding protein family [13], [14].This family consists of at least 25 different multifunctional proteins with molecular weights varying from 10–12 kDa with diversified functions ranging from regulation of cell cycle progression, transcription to protection from oxidative cell damage and apoptosis [13], [14]. S100B is primarily localized in astrocytes and is a hallmark of astrocytic activation in a fashion comparable to GFAP. A sudden opening of endothelial tight junctions causes a rapid elevation in serum S100B levels [11], [15], [16]. A low serum-to-cerebrospinal fluid (CSF) albumin ratio or an increase in serum S100B have been shown to have comparable sensitivity in detecting BBBD [17].

In contrast to markers of BBBD which are normally present in brain, indicators of brain damage are proteins synthesized in the CNS *after* a brain injury. Ubiquitin Carboxy-Terminal Hydrolase L1, NSE and GFAP correlate with brain damage; CSF and serum levels of these molecules had predictive value for traumatic brain injury [18]–[20]. However, since UCH-L1 and S100B are also increased after seizures [8], and given the fact that both have been reported to correlate with albumin CSF/serum ratio, the possibility exists that UCH-L1 also reports BBB integrity [21].

Human transferrin, a marker for alcohol consumption and abuse, is expressed at low levels in most adults and has 6 known transferrin isoforms (penta, tetra, tri, di, mono and asialo) [22]–[25]. The asialo, mono and disialo isoforms of transferrin are also known as carbohydrate-deficient transferrin (CDT). However a carbohydrate-free (desialated) isoform of transferrin is commonly known as beta-2 transferrin and is exclusively found in the cerebrospinal fluid (CSF) [26]. Upon CSF leakage, this protein can be found in blood, mucus and tears. Levels of this CSF protein in football players have never been measured and the impacts of sub-concussive head hits on this CSF leak marker are unknown.

The present study aims to identify neuronal injury after sub-CHI by monitoring changes in serum levels of UCHL1 and to determine the relationship between neuronal injury markers and a known marker of BBBD, S100B or to a CSF protein, beta-2 transferrin.

## Methods

### Ethics Statement

All patients signed an informed consent according to institutional review protocols at The Cleveland Clinic Foundation and the Declaration of Helsinki. Human research was conducted *as per* Institutional Review Board (IRB) guidelines (approved protocol at Cleveland Clinic IRB 4406 – PI Dr. Janigro; University of Rochester IRB 22809, 22971 – PI Dr. Bazarian). Players were selected among those participating to the local football college tournaments (Cleveland, OH). A written consent form was used to enroll players at the respective colleges. Players' demographic data (age, race, height, weight, history of previous concussion) were collected as per consent form and secured in a database available to the PIs (DJ/JB) only.

### Positive and Negative Controls

All volunteers or patients signed an informed consent according to institutional review protocols at the University of Rochester Medical Center and the Declaration of Helsinki. Positive controls were accrued from a prospective study of clinically-diagnosed mild TBI patients presenting to one of six emergency departments (ED) in western New York and northern Pennsylvania between 2008 and 2010. All patients provided blood sample within 6 hours of injury and underwent CT scanning. The mTBI study definition was adapted from the Center for Disease Control and Prevention’s definition 3 and consists of a blow to the head or rapid acceleration/deceleration resulting in at least one of the following: a loss of consciousness (LOC) <30 minutes, post-traumatic amnesia<24 hours, neuropsychological abnormality (any transient period of confusion, disorientation or impaired consciousness; in children<2 years old: irritability, lethargy or vomiting post-injury), or neurological abnormality (seizure acutely following injury, hemiplegia, or diplopia). In order to meet the mTBI definition, the subject must have a Glasgow Coma Scale (GCS) score of 13 or greater within 30 minutes of the injury.

Negative controls consisted of patients presenting to the University of Rochester Medical Center for routine blood-work. They were ineligible if they had a history of brain tumor, melanoma, or Alzheimer’s disease; a history of concussion, bone fracture or stroke within the prior month; or underwent surgery within the prior month.

### Blood Collection and ELISA Measurements (Football Players)

Players (n = 15) from a Northeast Ohio Varsity college team were enrolled. None of the players reported that they experienced a concussion. Blood samples were collected before and after two games in all 15 players. A total of 58 samples were collected instead of 60 due to 2 instances of a single player not being present at the pre-game blood draw. The number of head hits in all players was monitored by movie review and post-game interviews. Players' demographic data (age, race, height, weight, history of previous concussion) were collected as per consent form and secured in a database available to the PI (DJ) only. Demographic categories were collected to rule out confounding factors e.g., correlation between serum level of markers, race and body mass index (data not shown, see [10]).

Blood samples before and after games were collected in red cap Vacutainer sterile tubes (BD Bioscience) and the serum was separated by centrifugation (2000 RPM, 10 minutes). Samples were de-identified and assigned an internal ID. Serum samples were then stored at −80°C.

**Figure 1:**
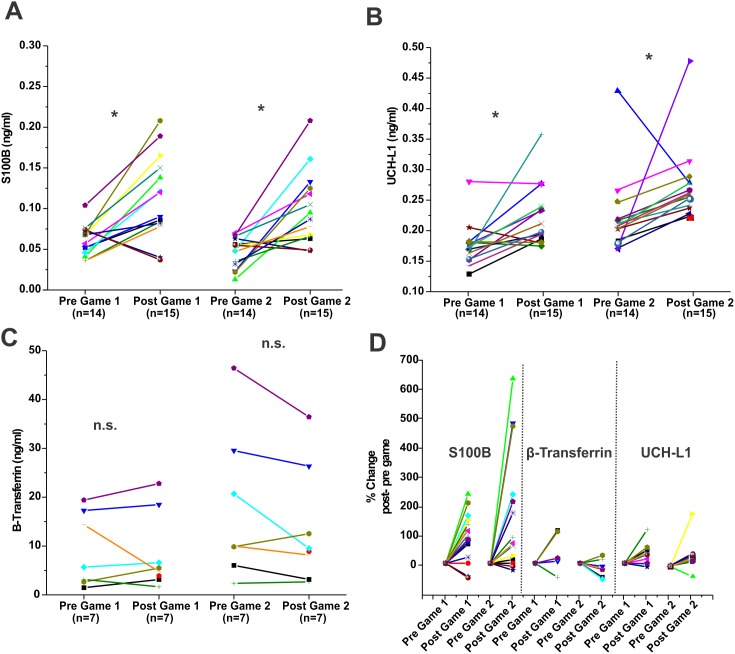
Pattern of S100B, UCH-L1 and β-2 transferrin changes in players after football games. Serum samples were drawn as described in the Methods. A total of 15 players were enrolled and analysis of samples reported here refers to two games played during the regular season. **A**)**, B**) and **C**) refer to absolute S100B, UCH-L1 and β-2 transferrin serum levels respectively. Serum levels were measured pre- (day before the game) and post-games (within one hour from the end of a game). In **D**) the normalized S100B, UCH-L1 and β-2 transferrin serum levels are shown side by side to allow a direct comparison. Normalized values for a given serum markers were obtained by the following equation: 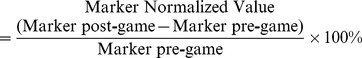. In the figures, each symbol represents a player and any given player is represented by the same symbol throughout this manuscript. Note that on average S100B and UCH-L1 were increased after a game, while the values for beta-2 transferrin remained unchanged. Also note that the beta-2 transferrin values pre-game were highly variable (**C**) while most of baseline values for S100B and UCH-L1 fell within a defined range. Statistical differences by student's t-test are shown as (*) for p<0.05; and *n.s.* for not significant.

**Figure 2:**
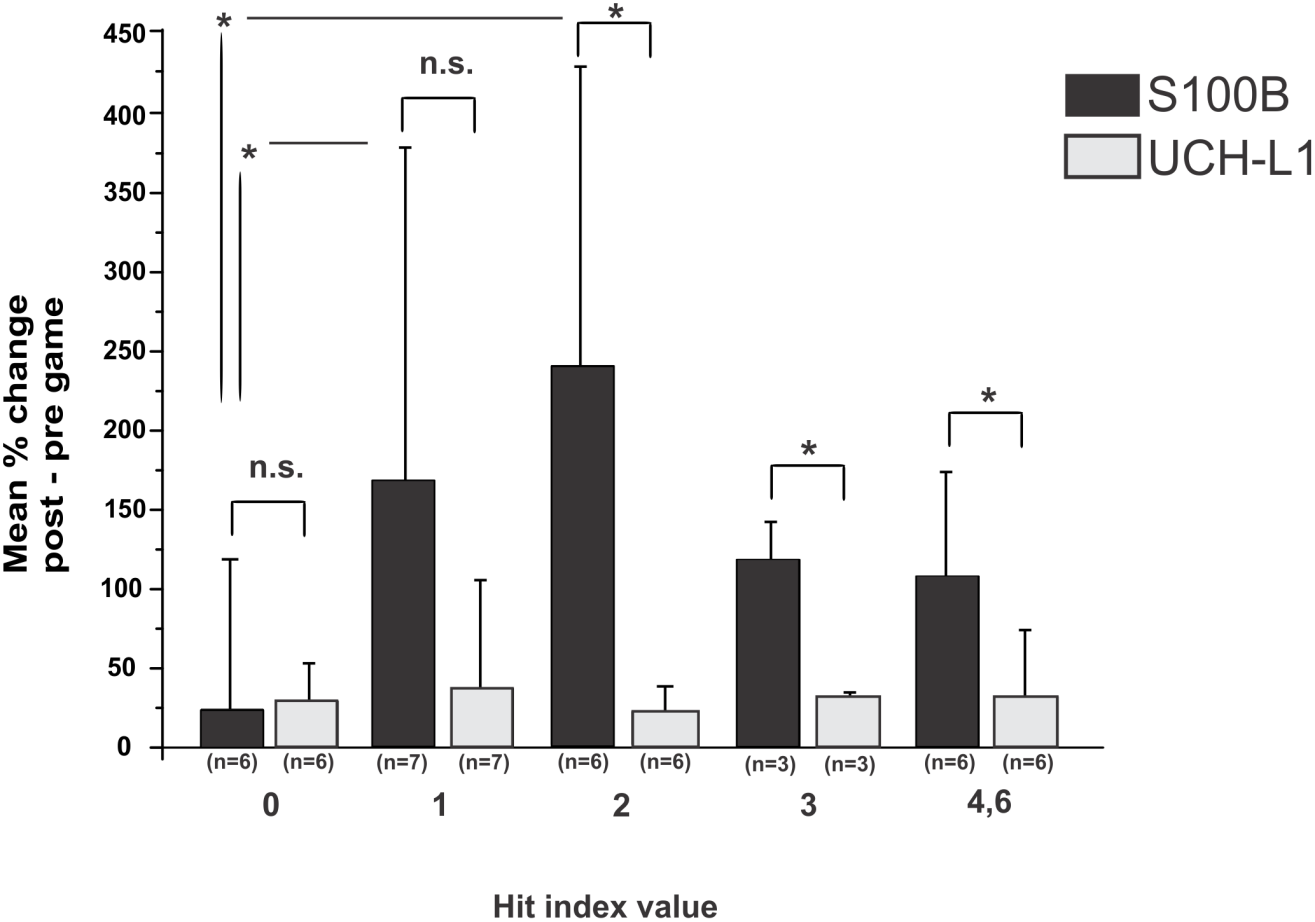
S100B serum surges correlate with the extent and number of head hits, while UCH-L1 does not appear to correlate with sub-concussive head hits. Mean percent change values of S100B and UCH-L1 levels (see equation (1)) were plotted against Head Hit Index (HHI) scores (see Methods). A statistically significant difference (by Wilcoxon Mann Whitney) was found between S100B surges at HHI of 0 and HHI of 1 and 2. UCH-L1 did not correlate with any of the HHI used for this study, and the levels of UCH-L1 measured after the game and normalized for their pre-game value as in (1) did not discriminate between a HHI of 0 (special team or played without any head hit) and any HHI (see also [10] for details on HHI). Statistical differences were analyzed by the Wilcoxon Mann Whitney test, both in the comparison between S100B and UCH-L1 at a particular HHI and in the comparison of the individual marker at a HHI 0 vs. HHI 1, 2, 3, and 4,6. To correct for type 1 error in multiple comparisons the Dunnett’s correction was used. Significance is shown as (*) for p<0.05; and *n.s.* for not significant.

**Figure 3:**
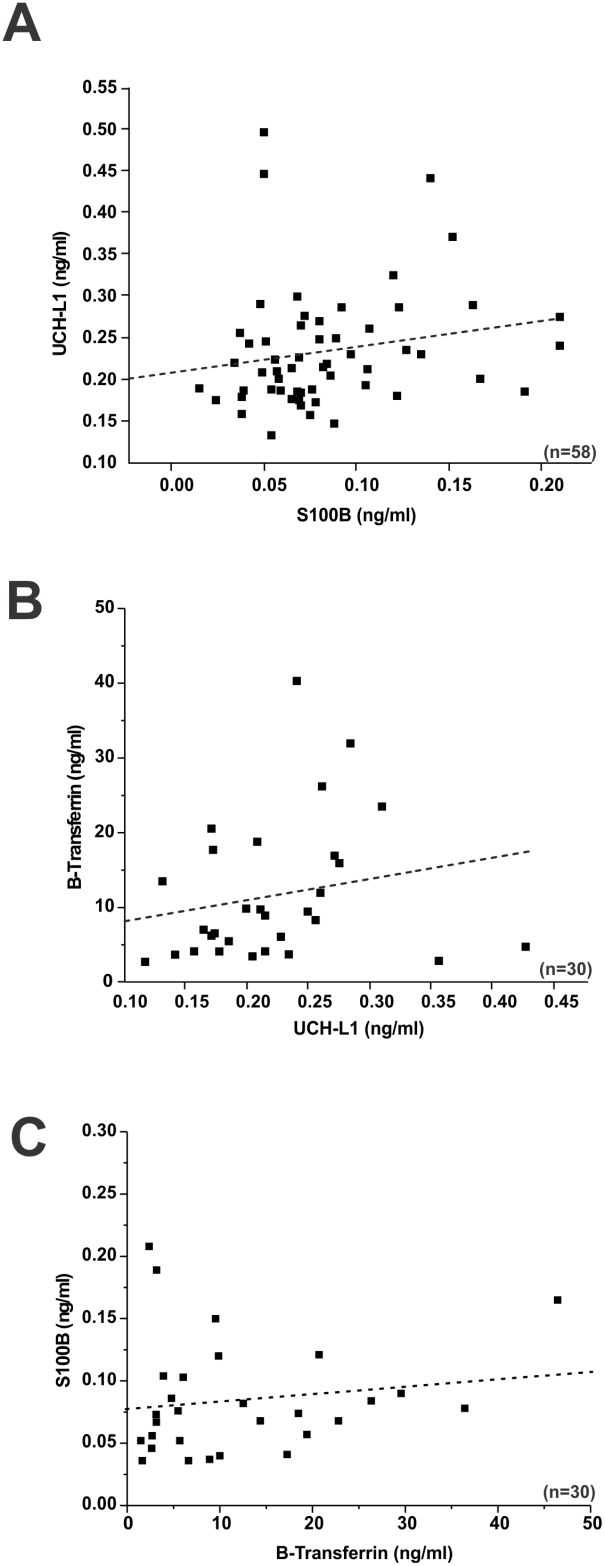
Serum levels of UCH-L1 do not correlate with S100B or levels of β-2 transferrin. **A**) S100B serum levels measured at pre- and post- game did not correlate with UCH-L1 serum levels (*p* = 0.16; R^2^ = 0.19). **B**) Lack of correlation between UCH-L1 or S100B and β-2 transferrin levels. β-2 transferrin serum levels measured at pre- and post- game (two games) plotted against UCH-L1 failed to show a statistically significant correlation (*p* = 0.28; R^2^ = 0.20). **C**) S100B serum levels measured at pre- and post- game (two games) did not correlate with β-2 transferrin serum levels (*p* = 0.22; R^2^ = 0.23). Significance is determined by ANOVA and denoted as (*) for p<0.05.

**Figure 4:**
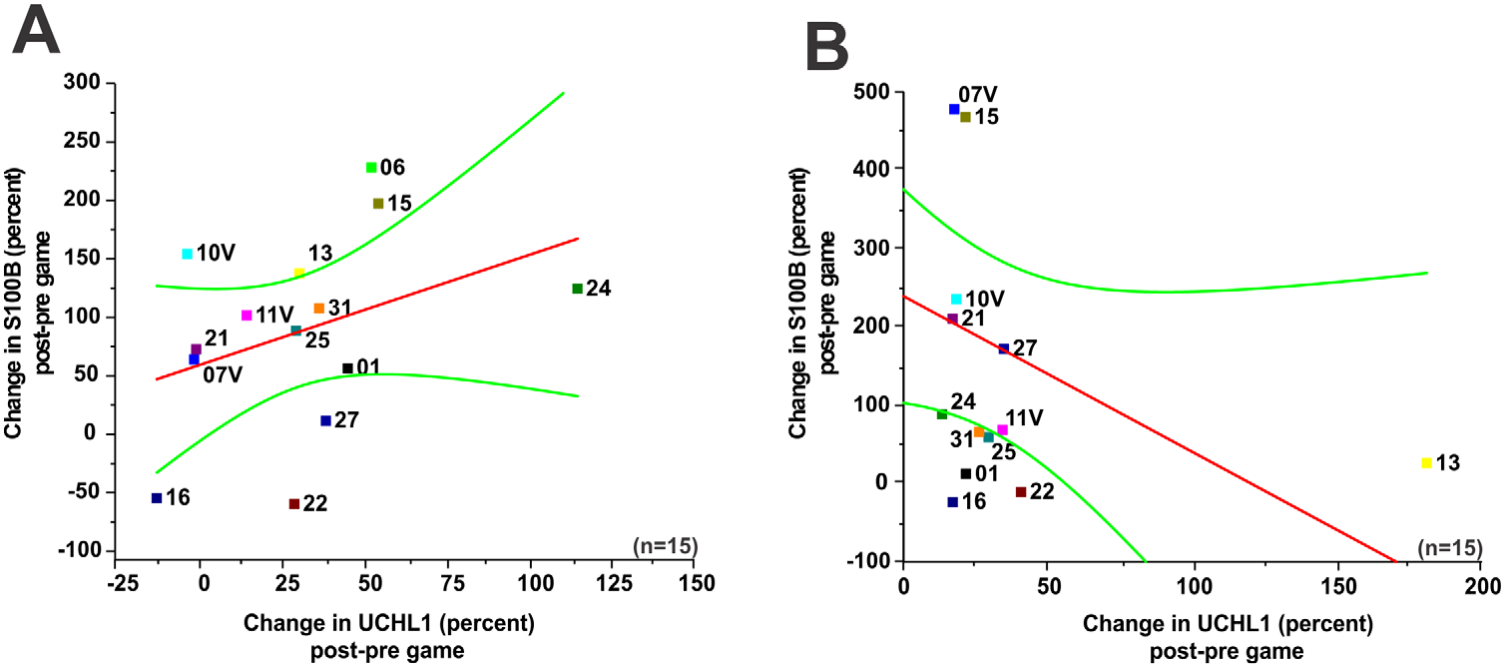
Markers’ change following a game confirms lack of correlation between S100B and UCH-L1. **A–B**) refers to normalized serum surges of S100B and UCH-L1 after game 1 and game 2. Note lack of correlation between these markers (*p* = 0.19; R^2^ = 0.37; game 1, *p* = 0.11; R^2^ =  −0.45; game 2). The *red line* is the linear regression fit while the outer lines show confidence intervals of 95%. We measured the normalized percent change in each game in an attempt to correct for the possibility of different measuring sensitivities for subconcussive head hits for the two markers measured by ELISA. Significance is determined by ANOVA and denoted as (*) for p<0.05.

**Figure 5:**
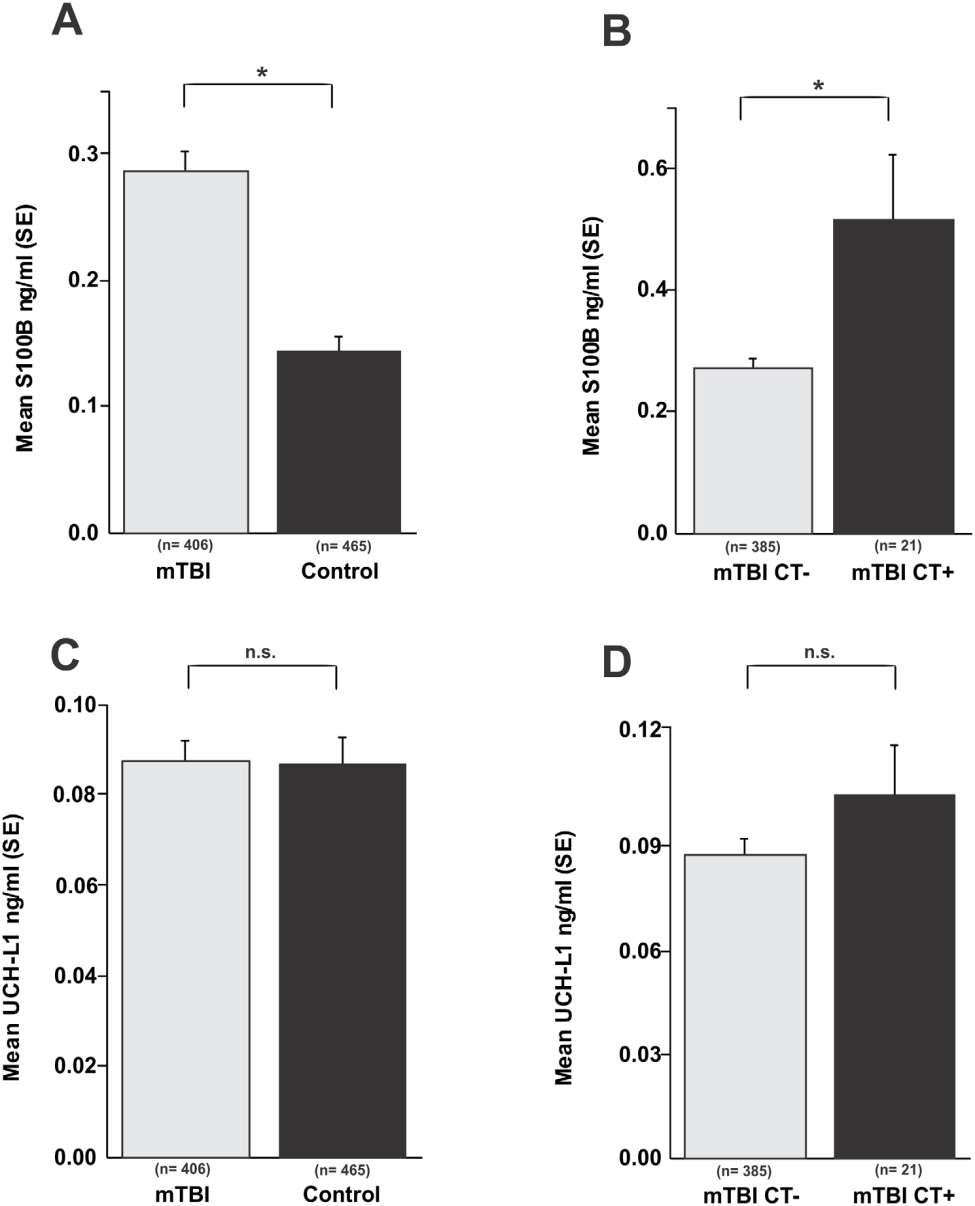
Lack of significant correlation between serum UCH-L1 levels and a diagnosis of mild TBI. **A**) S100B levels are significantly elevated in patients with a diagnosis of mild TBI compared to healthy controls (*p*<0.01) and, **B**) S100B levels correlate with post-traumatic findings on head CT (*p*<0.01). **C**) **and D**) UCH-L1 levels remain unchanged in patients with a diagnosis of mTBI compared to controls as well as in those patients with positive findings on head CT. Significance is determined by ANOVA and denoted as (*) for p<0.05.

S100B measurements (Figures 1–4) were performed by ELISA manufactured by Diasorin (Stillwater, MN). Note that for the experiments described in Figure 5 another detection method was used. 96 well plates were used and the analyte was sandwiched between two monoclonal antibodies directed against the beta-chain of the S100 dimer. Anti-human ELISA kits from Diasorin were read using a multi-plate fluorescent reader (at 450 nm). Fluorescent signals were converted into ng/mL *as per* standard curve concentrations. The process used is analogous to our previously published work [10], [15], [27]. The detection limit of this ELISA is 0.01 ng/ml. The intra-assay coefficient of variance of this test is around 6%.

A UCH-L1 ELISA kit from USCN (Houston, TX) and a beta-2 transferrin ELISA kit from Life Sciences advanced technologies (St. Petersburg, FL) were used to quantify UCH-L1 and beta-2 transferrin serum levels. The beta-2 transferrin ELISA had a detection limit of 0.01 ng/mL. The UCH-L1 ELISA had a range of detection between <0.056 and 10 ng/ml. Beta 2-transferrin was only measured in 8 players before and after two games. These 8 players were chosen based on highest and lowest serum S100B levels. The intra- and inter-assay coefficient of variance were <10% and >12% respectively.

### Blood Collection and ELISA Measurements (Control and mTBI Subjects)

Blood for S100B and UCH-L1 was drawn from participating control subjects and mTBI subjects within six hours of the time of injury. Four milliliters of whole blood was drawn into a serum separator tube and immediately placed on ice. Within 60 minutes, the blood was centrifuged at 3000 rpms for 10 minutes; the serum was aliquoted into 500ul tubes frozen at –80°C**.**

Serum S100B concentrations were determined by a fully-automatic electrochemoluminometric immunoassay (Elecsys S100; Roche Diagnostics, Penzberg, Germany) with detection limit of 0.005 and an upper range of 39 ng/mL. The analyte was sandwiched between two monoclonal antibodies directed against the beta-chain of the S100 dimer. Then, streptavidin-coated microparticles were added and the immunocomplex binds to the solid phase. In the measurement cell, unbound components were removed and a defined voltage used to initiate the electrochemiluminescent reaction. The resultant light emission was then measured using a photomultiplier.

UCH-L1 ELISAs were run in 96-well plates by standard methods with 20 l of serum run in duplicate, in each case made up to a final volume of 50 l with 2% non-fat milk in Tris-buffered saline with 0.1% Tween 20 (TBSt). Sample incubation and all antibody incubations were for 1 h with mild shaking at room temperature, with 100 l per well using 2% non-fat milk in TBSt as the diluent. The plates were extensively washed in TBSt between incubations using a BioTek ELISA plate washer. The UCHL1 ELISA makes use of a mouse monoclonal capture antibody and an affinity-purified rabbit detection antibody, followed by a commercial goat anti-rabbit antibody HRP conjugate (Sigma-Aldrich, St. Louis, MO). This ELISA has also recently been described (Lewis *et al.,* 2010). In each ELISA plate a standard curve was generated using recombinant human UCHL1, and signal was developed using 100 l per well of the TMB development solution of Pierce (Rockford, IL). The HRP reaction was run for 15 min at room temperature with mild shaking, and stopped with 50 l 1 M H_2_SO_4_ solution. Signal was measured at 450 nm absorbance using a BioTek Synergy plate reader.

### Game Film Review

Games were filmed using 4 cameras; videos were analyzed to detect and count head or soft tissue contacts. Two blinded operators performed film review. Intra-observed and inter-observer variation in film analysis was taken into account by comparing the data obtained by the two operators. Based on film analysis, players were grouped into the following categories: a) did not play (or played <5 minutes); b) played but did not experience any significant head hits or c) played and suffered repetitive head hits.

### Head Hit Index (HHI)

The HHI contains two separate measures, a self-reporting arm and an independent evaluation of hits (number and intensity) by post facto video review. A questionnaire was administered to each participant within 30 minutes following the completion of each game. HHI is not a tool to identify concussions but rather to quantify the occurrence of head hits. The questionnaire was designed by two doctors specialized in sport medicine. This questionnaire was intended to be feasible enough to administer to larger numbers of participants given the time and space limitations of a collegiate locker-room setting. The questionnaire included parameters previously recognized in the literature as possible important predictors in head injury and was developed with the aid of the authors and concussion experts [10]. Players enrolled in this study were asked to track the frequency and severity of significant collisions they experienced during each game. The questionnaire consisted of: 1) number of collisions experienced; 2) number of episodes of contact involving the head; 3) number of significant episodes of contact to the head; and 4) presence of acute symptoms (e.g., headache, neck pain, nausea). In the players enrolled in this study, no obvious loss of consciousness/awareness or acute traumatic episodes of dizziness were detected by the supervising team physicians during the events of interest. A Head Hit Index (HHI), ranging from 0 to 6, was derived from (a×b) where: a) is the number of head collisions and b) is the overall severity of these head hits (see Table 1). In the initial cohort of 29 players, some players were excluded from the analysis because of concussion related symptoms reported during pre-season games and because of persistent inconsistencies between film review results (several head hits observed by the two operators), self-reporting assessment (players did not report any experience of head hits) and blood S100B post-game elevations. These exclusions do not apply to the 15 players represented in this manuscript.

**Table 1:** Parameters of Head Hit Index (HHI) calculations.

HHI	A = 0	A = 1	A = 2	A = 3
(A×B)	(no hits)	(1–4 hits)	(5–20 hits)	(>20 hits)
**B = 0** **(e.g., negligible, body-helmet** **or ground-helmet contacts)**	0 - no head hits	0- negligible hits	0 - negligible hits	**N.A.**
**B = 1** **(e.g., player acknowledges the head** **hit)**	0 - no head hits	1 - e.g., a few head hitsduring game	2 - several butnormal hits	**3 - players who had>20** **normal hits**
**B = 1** **(e.g., player acknowledges the head** **hit)**	0 - no head hits	1 - e.g., a few head hitsduring game	2 - several butnormal hits	**3 - players who had>20** **normal hits**

A scoring system was used to segregate players based on the number and intensity of head hits experienced during games (see also Methods for details).

For the rest of the players film review results correlate with HHI scoring (*not shown*).

### Statistical Analysis

Origin 8.0 (Origin Lab, Northampton, MA, USA) and Jump 9.0 software were used for statistical analysis. The data was assessed for assumptions of normality and appeared to display a normal distribution in the population of pre- and post- game measurements during 2 games for the 15 players (Figures 1, 3, and 4) and thus parametric statistical tests were performed. Differences between pre- and post- game serum measurements (Figure 1) were analyzed by a paired student’s t-test. Due to the small sample size and possibility of non-normality, statistics were also analyzed using non-parametric tests and found to display similar results. Since no robust differences between parametric and non-parametric tests were seen we elected to use parametric statistical tests. The measures of head hit index for the 15 players during two games (a total of 30 measurements) were not normally distributed and non-parametric tests were performed. Data in Figure 2 was analyzed by the Wilcoxon Mann Whitney test, both in the comparison between S100B and UCH-L1 at a particular HHI and in the comparison of the individual marker at a HHI 0 vs. HHI 1, 2, 3, and 4,6. To correct for type 1 error in multiple comparisons in Figure 2 the Dunnet’s correction was used. In Figs. 3–5 which contain parametric variables, ANOVA was used to compare populations. Data points were fitted by a least square fitting routine. *R* is the square root of the coefficient of determination *R^2^*. In all figures, symbols with error bars indicate mean ± standard deviation (SD); *p<0.05 and was considered statistically significant and p values are shown in the figures or accompanying legends. n.s. represents not significant differences.

## Results

For enrollment of athletes at risk for sub-concussive head hits, we consented 15 Northeast Ohio collegiate football players. When studying patients with suspected TBI or healthy volunteers used as negative controls, only UCH-L1 and S100B were measured. The marker of CSF leakage, beta-2 transferrin, was not measured in trauma patients since a previous manuscript by one of us demonstrated that another marker of CSF barrier, transthyretin monomer, was not affected by mTBI [28].

Serum values for the marker of BBBD S100B were, on average, elevated after a game (Figure 1A). Significant elevations in S100B levels after game 1 (pre-game 1: mean 0.061±0.019 ng/ml; post-game 1: mean 0.113±0.052 ng/ml, as well as after game 2 (pre-game 2: mean 0.046±0.019 ng/ml; post-game 2: mean 0.100±0.045 ng/ml) were observed. A similar pattern of post-game elevation was seen for the putative marker of TBI, UCH-L1 (B), (Game 1; pre-game 1: mean 0.182±0.037 ng/ml; post-game 1: mean 0.246±0.074 ng/ml. Game 2; pre-game 2: mean 0.230±0.068 ng/ml; post-game 2: mean 0.282±0.065 ng/ml). Beta-2 transferrin levels were highly variable across subjects and unaffected by games (C), (Game 1; pre-game 1: mean 9.14±7.61; post-game 1: mean 8.35±7.81 ng/ml. Game 2; pre-game 2: mean 17.85±15.63 ng/ml; post-game 2: mean 13.46±11.84 ng/ml). To account for different absolute values of these markers, figure 1D shows a side-by-side comparison of their behavior normalized for their pre-game levels. Note that both UCH-L1 and S100B were elevated in most but not all players (S100B: mean % change game 1 90.3%, mean % change game 2 165.4%; UCH-L1: mean % change game 1 30.5%, mean % change game 2 31.3%, SD 46.7). Since the goal of this study was to correlate sub-concussive hits to appearance of serum markers, we attempted to identify causality between the former with the latter. We plotted changes in UCH-L1 or S100B against a “hit index” (see table 1).

Figure 2 shows a correlation between S100B levels (reported as % change (post-game - pre-game)/pre-game) and number/severity of sub-concussive head hits. UCH-L1 levels are reported in an identical manner. The consolidation of groups 4 & 6 was performed in order to obtain a sample size large enough to perform statistics (n = 4 for HHI of 4, n = 2 for HHI of 6). When the S100B and UCH-L1 were compared, a significant difference between S100B and UCH-L1 increases related to a given head hit index value (HHI values 2, 3, and 4,6) was seen. In contrast, there was no correlation between head hits and UCH-L1 levels.

Levels of S100B and UCH-L1 were then cross-compared to determine a possible correlation between these two markers independent of sub-concussive head hits. This was achieved in two ways. We first compared all serum measurements in football players (Figure 3). S100B serum levels did not correlate with UCH-L1 serum levels (*p* = 0.16; R^2^ = 0.19). In addition, beta-2 transferrin serum levels did not correlate with either UCH-L1 (*p* = 0.28; R^2^ = 0.20) or S100B (*p* = 0.22.; R^2^ = 0.23).

We also wished to determine if a hidden relationship between S100B and UCH-L1 may be unveiled by an analysis taking into account values from individual players measured before and after a given game (Figure 4). In other words, we wished to determine whether consistent post-game changes in both markers existed in a given player. When changes in S100B and UCH-L1 measured after each of the two games were plotted and the players singled out, again no correlation was found (game 1 p = 0.19; R^2^ = 0.37, game 2 p = 0.11; R^2^ =  −0.45). Note also that while in game 1 an apparent positive, yet statistically not significant, correlation was found, in the following game a negative, but again not statistically significant, correlation was observed. Taken together, these results show a lack of correlation between S100B and UCH-L1.

The results presented so far show a lack of correlation between serum reporters of blood-brain barrier disruption and markers of brain injury in non-concussed football players. Since UCH-L1 is considered to be a marker of brain damage in clinical TBI [8], [19], [29], we wished to investigate a possible correlation between these markers in a population with suspected or confirmed TBI (Figure 5). Note that S100B segregated patients with a documented TBI from controls (*p*<0.01; Figure 5A); in addition, S100B was elevated in patients with TBI and a measurable radiological consequence of TBI (CT+) compared to patients with a good prognosis (normal CT; CT- in the Figure 5B). This difference was statistically significant (*p*<0.01). In contrast, UCH-L1 failed to accurately identify patients with TBI compared to control (Figure 5C), or to predict TBI severity measured by CT (Figure 5D).

## Discussion

The main finding of this manuscript is the apparent lack of clinical significance of the marker of brain damage UCH-L1. We used two different immunoassays for each marker and three distinct cohorts to study the behavior of serum markers after subconcussive head hits; these were football players, patients with a diagnosed traumatic brain injury and control subjects. We used S100B as a marker of blood-brain barrier integrity and the asialo form of transferrin (beta-2 transferrin) as a marker of blood-to-CSF barrier. The latter was, as expected, virtually unaffected by subconcussive events, while S100B levels, but not UCH-l1, directly correlated with subconcussive head hits and traumatic brain injury. These results are in sharp contrast with findings by others related to UCH-L1 [30], but confirm that S100B is a sensitive marker of subconcussive head hits and clinical mTBI.

### Football-related Subconcussive Head Hits

The results obtained in football players were intentionally designed to provide evidence in support or against the use of UCH-L1 as an aid to diagnose subconcussive or concussive injuries in athletes as there is no validated test to diagnose subconcussion. A larger study focused on BBBD and S100B [10] has been recently published. The goal of this study was to show for the first time how, in athletes, a marker of CNS damage (UCH-L1) correlates with a marker of BBBD (S100B). The results showed no correlation between these two markers, nor did they reveal any correlation between UCH-L1 and HHI indices. In addition, UCH-L1 failed to correlate with data obtained from clinical mTBI. The difference in measured ranges for S100B and UCH-L1 between football players and clinical mTBI patients can be attributed to the different methods employed to measure these biological serum markers (see Methods).

Our results are at odds with previous findings (reviewed in a recent paper [19]). To our knowledge there are no previous reports of normal serum values for UCH-L1 in healthy young adults since most of the previous work deals with TBI patients [21], [30]. In the present study control subjects had UCH-L1 levels comparable to pre-game football players (on average around 0.1 ng/mL). These values were similar to what measured in mTBI patients regardless of positive or negative findings on CT. Previous efforts produced conflicting results on the significance of UCH-L1 in TBI: on the one hand, it was shown that UCH-L1 correlates with neuronal damage [18], [30] while Bazarian *et al* [21] have shown that in a similar population of patients this protein correlated with serum/CSF indices of blood-brain barrier disruption. In our cohort of players, UCH-L1 did not correlate with either variable, being increased after a game independent of levels of S100B or HHI. Admittedly, our use of TBI patients as positive control may be problematic given the fact that ideally we should have used concussed football players. However, results comparing concussed vs. non-concussed players were published elsewhere [31] to confirm the rationale of using TBI as a positive control in this small sample study.

Since UCH-L1 is also expressed by other cell types, it is possible that the game of football caused an increased serum level owing to extracranial events [32] A reasonable explanation for “football-related” UCH-L1 surges derives from the finding that this protein is expressed by presynaptic neurons at the neuromuscular junction [33]. If this hypothesis is correct, the impact of repeated traumatic hits to the body may cause release of neuromuscular UCH-L1. The kinetics of UCH-L1 is not well understood. It is unclear how quickly this protein rises after injury although in a previous study we found it was elevated 12 hr after injury [21]. Thus low UCH-L1 levels within 1 hour of the end of a game could reflect that it has been released by injured neurons but has not yet risen to a detectable level in serum. Under these conditions, a correlation with head hits or blood-brain barrier disruption is not expected. An alternative explanation links release of this protein by inflammatory cells [34]. In this study, we attempted to correlate UCH-L1 levels with IL-6, thyroid stimulating hormone, and C-reactive protein. No relationships were found between UCH-L1 and these serum markers (*data not shown*). It is therefore unlikely that UCH-L1 correlates with systemic inflammation.

In conclusion, our results have shown that UCH-L1, previously defined as a marker of TBI, lacks association with traumatic head injury or sub-concussions. In addition, UCH-L1 failed to correlate with blood-brain barrier disruption measured in football athletes by indrect serum-based assays. The clinical value of UCH-L1 appears to be limited to severe head injury where significant CSF elevation may improve its gradient to promptly extravasate in serum across a disrupted BBB.
